# Vector competence of European *Aedes* mosquito species for Japanese encephalitis virus under fluctuating temperature conditions

**DOI:** 10.1016/j.crpvbd.2025.100302

**Published:** 2025-07-31

**Authors:** Anna M. Ciećkiewicz, Julia Ettlin, Eva Veronesi, Andrea Marti, Obdulio Garcia-Nicolas, Jeannine Hauri, Artur Summerfield, Alexander Mathis, Niels O. Verhulst

**Affiliations:** a National Centre for Vector Entomology, Institute of Parasitology, Vetsuisse and Medical Faculty, University of Zürich, Switzerland; b Institute of Virology and Immunology IVI, Mittelhäusern, Switzerland; c Institute of Microbiology, Department of Environment Constructions and Design, University of Applied Sciences and Arts of Southern Switzerland (SUPSI), Mendrisio, Switzerland

**Keywords:** JEV, Japanese encephalitis, Flavivirus, Arbovirus, *Aedes*, Vector competence, Zoonosis

## Abstract

The Japanese encephalitis virus (JEV) is a mosquito-borne flavivirus endemic to much of Asia and the Western Pacific, both temperate and tropical regions. Globalisation and the expansion of invasive mosquito species raise concerns about their potential establishment in Europe and other currently non-endemic regions. However, limited knowledge exists regarding the vector competence of European mosquitoes, particularly under the region’s characteristic fluctuating temperatures. While *Culex* species are primary JEV vectors, the role of *Aedes* mosquitoes remains unclear. This study assessed the vector competence of field-caught or low-generation colony-derived *Aedes albopictus*, *Ae. japonicus*, and *Ae. vexans* from Switzerland under a fluctuating temperature regime (16–28 °C), using *Culex quinquefasciatus* as a reference. Mosquitoes were exposed to JEV genotype I-b and incubated for 7 and 14 days. RT-qPCR was used to analyse mosquito body parts and saliva to determine infection, dissemination, transmission rates and transmission efficiency. *Aedes albopictus*, *Ae. japonicus*, and *Cx. quinquefasciatus* were competent vectors. *Aedes japonicus* showed the highest infection rate (13.6%, 9/66) compared to *Ae. albopictus* (3.0%, 3/101) and *Cx. quinquefasciatus* (6.9%, 7/101), while *Ae. vexans* was refractory (0/80). Dissemination was observed in *Ae. japonicus* already 7 days post-exposure, preceding other species. *Aedes japonicus* had the highest transmission rate (66.7%, 2/3) and efficiency (6.1%, 2/33). This study demonstrates that European *Aedes* mosquitoes can serve as JEV vectors under fluctuating temperatures and may contribute to virus transmission despite being considered secondary vectors. The findings emphasise that species-specific assessments under realistic temperatures are essential in evaluating the risk of JEV establishment in temperate regions.

## Highlights

• *Aedes* mosquitoes from Switzerland were tested for Japanese encephalitis virus (JEV) under fluctuating temperatures.
• *Aedes japonicus* was the most efficient vector, with JEV dissemination after 7 days.
• *Aedes albopictus* was a competent JEV vector after 14 days of incubation.
• *Aedes vexans* was refractory to the infection with JEV.

## Graphical abstract

**Figure:**
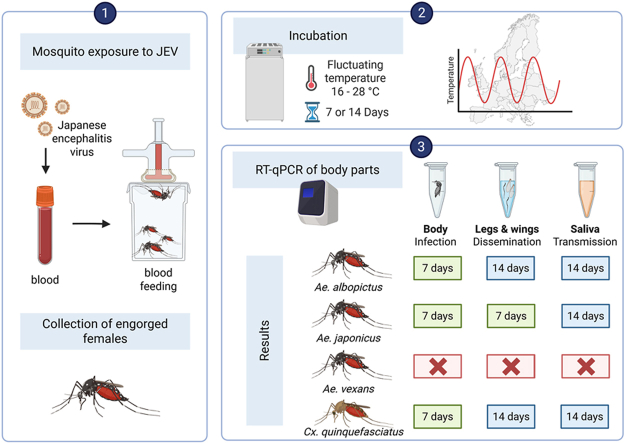

## 1 Introduction

Mosquito-borne diseases are a growing public health concern with significant socio-economic impact, particularly through zoonotic transmission and livestock losses. Human activities, such as international trade and travel, facilitate the introduction of arthropod-borne viruses (arboviruses) and vectors to new geographical locations and immunologically naïve host populations (Jones et al., 2008; Pierson and Diamond, 2020). Additionally, the northward expansion of (sub-)tropical mosquito species, driven by climate change, contributes to the growing global burden of vector-borne diseases (Messina et al., 2019; Nakase et al., 2024). Recent outbreaks, including those of West Nile virus (Lu et al., 2024) and dengue virus (Cassaniti et al., 2023) in Europe, highlight the need for a better understanding of vector roles in arbovirus transmission cycles to develop region-specific outbreak response strategies.

Japanese encephalitis virus (JEV) is a mosquito-borne flavivirus endemic across much of Asia and Oceania, with seasonal recurrence in temperate regions of East Asia associated with the presence of the vectors (Schuh et al., 2013, 2014). JEV has recently been detected outside its endemic regions, including a major outbreak in Australia in 2022 (Mackenzie et al., 2022; Furlong et al., 2023). In 2018, JEV RNA was identified in a *Culex* mosquito pool in Xinjiang, China (Hameed et al., 2021a), and in 2012, in a *Culex pipiens* pool in Italy, raising concerns about its potential spread into Europe (Ravanini et al., 2012).

JEV persists in an enzootic cycle involving birds and pigs as reservoir hosts. The high viremia levels in these hosts enable mosquito infection during blood meals and, subsequently, the virus’ transmission (Weaver and Barrett, 2004; Mansfield et al., 2017; Hameed et al., 2021b). Additionally, vector-free transmission has been experimentally proven in pigs, adding further complexity to the understanding of the JEV transmission cycle (Ricklin et al., 2016). In contrast, cattle, horses and humans are considered dead-end hosts. They experience varying levels of disease severity, including encephalitis and long-term neurological sequelae, but do not transmit the virus further due to insufficient viremia (Ilkal et al., 1988; Mansfield et al., 2017). Each year, an estimated 100,000 clinical cases of Japanese encephalitis occur in humans, with children in endemic regions being the most affected (Quan et al., 2020).

A wide range of mosquito species from the endemic regions, particularly those within the genera *Culex* and *Aedes*, have demonstrated vector competence for JEV, defined as the ability of the mosquito to become infected, sustain the infection and transmit the virus (reviewed by Auerswald et al., 2021; Van den Eynde et al., 2022). Significant variations in competence exist across genera and species within the same mosquito genus (Kain et al., 2022). *Culex tritaeniorhynchus* and *Cx. annulirostris* are considered primary vectors in Asia and Australia, respectively, due to their zoophilic feeding patterns, frequent isolation from field-caught mosquitoes in JEV-endemic regions and proven vector competence in laboratory studies (Hall-Mendelin et al., 2012; Su et al., 2014). Although JEV is rarely found in *Aedes* mosquito species in the field, laboratory experiments consistently demonstrate their vector competence (Su et al., 2014; de Wispelaere et al., 2017; Faizah et al., 2020). Even with low transmission rates in the field, *Aedes* species may contribute to maintaining the infection in endemic regions through horizontal and vertical transmission (Takashima and Rosen, 1989). Additionally, their high abundance and opportunistic feeding behaviour may facilitate transmission to dead-end hosts, particularly in peri-urban and rural areas near pig farms (Van Den Hurk et al., 2003a; Hall-Mendelin et al., 2012). This emphasises the need to consider their role alongside primary vectors in understanding JEV ecology.

Predicting epidemiological patterns of arboviruses poses challenges due to complex host-pathogen interactions and vector biology, further complicated by environmental factors, such as temperature fluctuations (Lambrechts et al., 2011; Carrington et al., 2013). Additionally, recent reports show variations in mosquito populations’ susceptibility to the same pathogen across different regions (Hernández-Triana et al., 2022). While temperature plays a key role in mosquito survival, behaviour, immunity and virus transmission (Adelman et al., 2013; Winokur et al., 2020; Hug et al., 2024), previous laboratory studies have been conducted mainly under constant temperatures, limiting epidemiological risk assessment in the local context in temperate regions.

Due to the limited number of studies investigating the competence of European mosquitoes for JEV, this study aims to assess the vector competence of *Aedes* species locally abundant in Switzerland. Although *Culex* species are primary JEV vectors, our focus shifts to secondary vectors to better understand their potential role in JEV ecology, as they may prove significant under certain ecological settings. Moreover, we applied relevant temperature regimes for temperate regions in our experimental design and used field-caught or low-generation colony-derived *Aedes* mosquitoes. Mosquitoes were exposed to JEV and incubated in fluctuating temperatures representative of a Central European summer. The findings reveal species-specific differences in vector competence and provide valuable insights into the risk of JEV establishment in Europe.

## 2 Materials and methods

### 2.1 Virus origin and quantification

The JEV genotype I-b (GI-b) (strain CNS769_Laos_2009; kindly provided by Dr. Charrel, Aix-Marseille Universite, Marseille, France) used in this study was isolated from a human patient in Vientiane, Laos (Aubry et al., 2013). Virus stock was propagated three times on *Aedes albopictus* clone C6/36 cell line (ATCC) as described by Marti et al. (2024).

The infectious virus was quantified by viral titration in C6/36 cells cultured at 28 °C and 5% CO_2_. Briefly, the 96-well plates were seeded with 1 × 10^5^ cells per well and incubated for 24 h. Once confluent, the cells were inoculated with the JEV sample serially diluted ten-fold, starting dilution 1:10, in minimal essential medium (MEM, Gibco #31095-029, Thermo Fisher Scientific, Reinach AG, Switzerland), supplemented with 1% MEM non-essential amino acid solution (100×) (MEM NEAA, Gibco #11140-35, Thermo Fisher Scientific), 1% sodium pyruvate 100 mM (NaPy, Gibco #11360-039, Thermo Fisher Scientific) and 2% foetal calf serum (FCS, BioConcept). A total of 200 μl of each dilution was added to the wells in quintuplicate and incubated at 28 °C and 5% CO_2_ for three days. The infected cells were stained following the Immunoperoxidase Monolayer Assay (IPMA), as previously described (Ricklin et al., 2016). The titre was expressed as 50% tissue culture infectious dose per millilitre (TCID_50_/ml) and calculated to be 4.56 × 10^7^ TCID_50_/ml according to the Reed-Muench method (Reed and Muench, 1938).

### 2.2 Mosquito origin and rearing

The study involved three European *Aedes* mosquito species and a tropical *Culex* mosquito as a reference species. *Aedes japonicus* eggs were collected with ovitraps in the Schwamendingen Cemetery, Zürich, Switzerland (47°24′5″N, 8°34′28″E) following a previously established protocol (Glavinic et al., 2020), while *Aedes vexans* eggs were collected from moss in known breeding sites in Thurauen, Switzerland (47°35′37"N, 8°35′19"E). Both species were reared to adulthood and used directly in the experiments.

*Aedes albopictus* and *Cx. quinquefasciatus* mosquitoes used in the study were colony-derived. *Aedes albopictus* originated from Ticino, Switzerland, was kindly provided by the Scuola Universitaria Professionale della Svizzera Italiana (SUPSI), and was maintained at our Institute for up to 5 generations. *Culex quinquefasciatus* originated from California, USA, was provided by the Institut de Recherche pour le Développement (IRD) in Montpellier, and the colony was originally established in 1966 (Georghiou et al., 1966). Eggs of all *Aedes* species were collected during the summer of 2019.

*Aedes* species eggs laid on the germination paper within ovitraps were submerged in deionised water to induce hatching. Moss containing *Ae. vexans* eggs were placed in a plastic bucket, filled with deionised water to a level of 10 cm, and incubated at room temperature until larvae emerged. *Culex quinquefasciatus* eggs were collected in white plastic cups filled with deionised water and transferred to rearing trays three times a week. Larvae and pupae of both field-collected mosquitoes and those maintained in colonies were kept in plastic 5-L buckets filled with deionised water, and Tetramin® (Tetra GmbH, Melle, Germany) was provided as a source of nutrition. Upon emergence, adults were transferred into cube-shaped cages (32.5 × 32.5 × 32.5 cm, Bugdorm 43030F, MegaViewScienceCo. Ltd., Taichung, Taiwan) and provided with 10% sucrose solution *ad libitum*. Adult mosquitoes used for colony maintenance were fed on bovine blood provided by a local slaughterhouse (SBZ Schlachtbetrieb Zürich AG, Switzerland) with EDTA as an anticoagulant using a Hemotek system (Hemotek Ltd., Lancashire, UK) with an artificial membrane (Parafilm M, Sigma-Aldrich, Buchs, Switzerland). The insects were kept under a 16 h:8 h light:dark cycle, including 1 h dusk and dawn, at 24 ± 0.5 °C with 65 ± 5% relative humidity (RH) for *Ae. japonicus*, *Ae. vexans*, and at 27 ± 0.5 °C with 75 ± 5% RH for *Ae. albopictus* and *Cx. quinquefasciatus*.

### 2.3 Mosquito exposure to JEV

Mosquitoes were exposed to a blood meal spiked with JEV. The infectious blood meal consisted of heparinised bovine blood mixed with JEV cell culture supernatant (1:9), yielding a final titre of 4.56 × 10^6^ TCID_50_/ml, and supplemented with phagostimulant (ATP at 5 mM; SIGMA A2383). In pigs, the amplifying hosts of JEV, genotype I-b infection induces peak viremia levels of approximately 10^5^–10^6^ TCID_50_/ml (Park et al., 2021; Marti et al., 2024).

Mosquitoes aged 7–12 days were deprived of sugar for 24 h (*Ae. albopictus* and *Ae. vexans*) or 48 h (*Ae. japonicus* and *Cx. quinquefasciatus*) before being exposed to the infectious blood meal. Female mosquitoes were aspirated from rearing cages and placed in 500 ml plastic containers (20 females per container), with the tops covered by netting and socks worn overnight as attractants. Through the net, they were exposed to the Hemotek feeding system, as described above, containing 3 ml of infectious blood heated to 37 °C. Light exposure was reduced during feeding of both *Culex* and *Aedes* species, and mosquitoes were further stimulated with dry ice as a CO_2_ lure.

After 1 h of feeding, mosquitoes were anesthetized at −20 °C for 4–6 min and placed on a Petri dish on a cold plate for the selection of blood-fed females. Only fully engorged females were used for further procedures. Up to three mosquitoes were collected on the day of the exposure to the blood meal (Day 0), immediately frozen at −80 °C and stored as a baseline and positive control. The remaining mosquitoes were moved into netted cardboard cylinders (12 cm diameter, 15 cm height, max of 80 females per container) and provided with a 10% sucrose solution *ad libitum* on a cotton ball. They were incubated in a climate chamber (MLR-352H, Panasonic, Ltd., Japan) for 7- or 14-days post-exposure (dpe) under a fluctuating temperature regime of 16–28 °C and constant 80% humidity (Supplementary file 1: Table S3), reflecting summer conditions in northern Switzerland (www.meteoswiss.admin.ch), with the photoperiod as described above.

### 2.4 Harvesting mosquito body parts and saliva collection

Alive mosquitoes were anesthetized as described above and placed on a glass slide to remove legs and wings. Forceps were autoclaved between experiment groups and disinfected between individuals with Virkon™ (Virkon S, Lanxess) and 70% ethanol, and a new glass slide was used for each mosquito. Saliva was collected by inserting the proboscis of leg- and wing-deprived females into a 20 μl pipette tip containing 5 μl FCS for 30 min. The body parts and saliva solution were collected in 1.5 ml tubes (#72.706, Sarstedt, Germany) containing 45 μl (for saliva) or 100 μl (for legs and wings) of Dulbecco’s modified eagle medium (DMEM, Gibco #31885-023, Thermo Fisher Scientific), supplemented with 2% FCS. The bodies were stored dry. Samples were immediately frozen at −80 °C.

### 2.5 Virus RNA detection

**Fig. 1:**
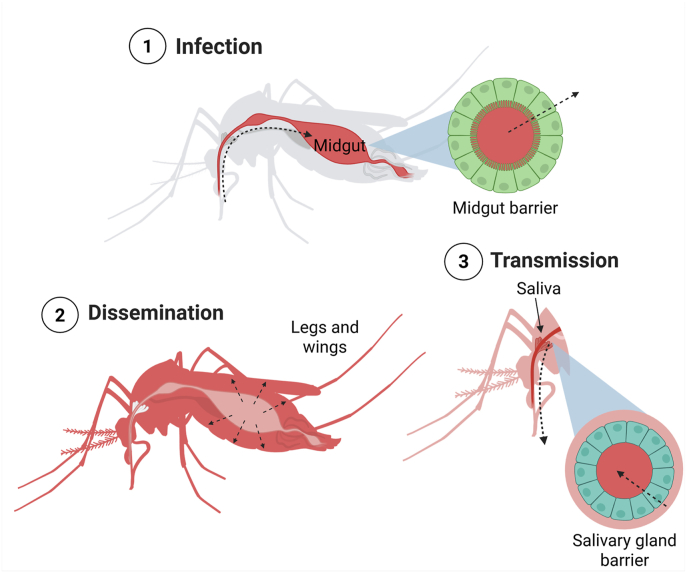
Schematic representation of JEV dissemination in a mosquito (dotted arrows), including key physiological barriers (midgut, salivary gland barriers); red colour indicates virus location in the mosquito body.

The presence of JEV RNA was tested in duplicates by reverse transcription-quantitative polymerase chain reaction (RT-qPCR). First, mosquito bodies were screened for infection. The legs and wings of infected mosquitoes were then tested to confirm virus dissemination, followed by testing saliva from mosquitoes with disseminated infection to test for virus transmission capacity (Fig. 1).

Before homogenisation, 100 μl of DMEM (2% FCS) was added to the body samples. Mosquito bodies, legs and wings were homogenised using a TissueLyser® II (Qiagen, Hilden, Germany) at 25 Hz for 2 × 30 s, using one 3.2 mm stainless steel bead per sample (Fisher Scientific, Reinach, Switzerland). After processing, 900 μl of additional DMEM (2% FCS) was added, the bead was removed with a decontaminated magnet, and the samples were centrifuged. A 140 μl of the body parts homogenate or 5 μl of saliva sample mixed with 135 μl of sterile Dulbecco’s phosphate-buffered saline (DPBS (1×), #14040-091-500 ML, Gibco) was used directly for viral RNA extraction with the QIAmp® Viral RNA Mini kit (#52906, Qiagen, Hilden, Germany), performed according to the manufacturer’s instructions. The elution buffer volume was reduced to 45 μl for higher RNA concentration.

RT-qPCR was conducted using the iTaq Universal Probes One-Step Kit (#1725141, Biorad). Primers and probe (Microsynth, Balgach, Switzerland) were as follows: 5′-ATC TGA CAA CGG AAG GTG GG-3′ forward primer; 5′-TGG CCT GAC GTT GGT CTT TC-3′ reverse primer; 5′-FAM-AGG TCC CTG CTC ACC GGA AGT-TAMRA-3′ probe (Marti et al., 2024) with a final concentrations of 800 nM and 200 nM for primers and probe, respectively. The 20 μl reactions included 5 μl of RNA template. Thermo-cycling conditions were reverse transcription at 50 °C for 10 min, polymerase activation and cDNA denaturation at 95 °C for 3 min, followed by 45 cycles of 95 °C for 15 s (denaturation) and 60 °C for 30 s (annealing and elongation). Reactions were performed in duplicates using a CFX96 Real-Time System (Biorad, Basel, Switzerland). To minimise the likelihood of false positives and ensure the reliability of the RT-qPCR reactions, samples were considered positive for further data analysis if both duplicates had a cycle of threshold (Ct) value of 35 or lower.

### 2.6 Biosafety

All the procedures involving infectious JEV and infected mosquitoes were performed in a biosafety level 3 laboratory of the Laboratory Animal Service Centre at the University of Zurich. The staff members were vaccinated against JEV.

### 2.7 Data analysis

The rates of infection (IR), dissemination (DR), and transmission (TR), as well as dissemination (DE) and transmission efficiencies (TE), were assessed in the four mosquito species at 7 and 14 days post-exposure to JEV. IR indicates the percentage of females that tested positive for JEV RNA in their bodies. DR and DE represent the percentage of females with disseminated infection, as determined by JEV RNA detection in legs and wings, calculated among infected females and all females, respectively. TR and TE represent the percentage of females with JEV RNA in saliva, relative to those with disseminated infection and all females, respectively.

A generalized linear model (GLM) with a logit link function was applied to analyse JEV presence in mosquito bodies (binomial outcome: JEV RNA detected [1] or not detected [0]). The categorical explanatory variables were incubation period (2 levels: 7 or 14 dpe) and mosquito species (4 levels: *Ae. albopictus*, *Ae. japonicus*, *Ae. vexans*, *Cx. quinquefasciatus*). When the GLM showed significance, Fisher’s exact test based on a 2 × 2 contingency table was used to evaluate statistical differences in IR within the significant factor. Due to small sample sizes, no statistical analysis was performed for the DR, DE, TR and TE.

The figures were created using Graph Pad Prism 10.2.3 (GraphPad Software, La Jolla, USA). Statistical analysis was performed using R (v4.4.2) (R Core Team, 2025) using the *tidyverse* (Wickham et al., 2019), *ggfortify* (Tang et al., 2016) and *car* (Fox and Weisberg, 2018) packages.

## 3 Results

### 3.1 Infection rate

**Fig. 2:**
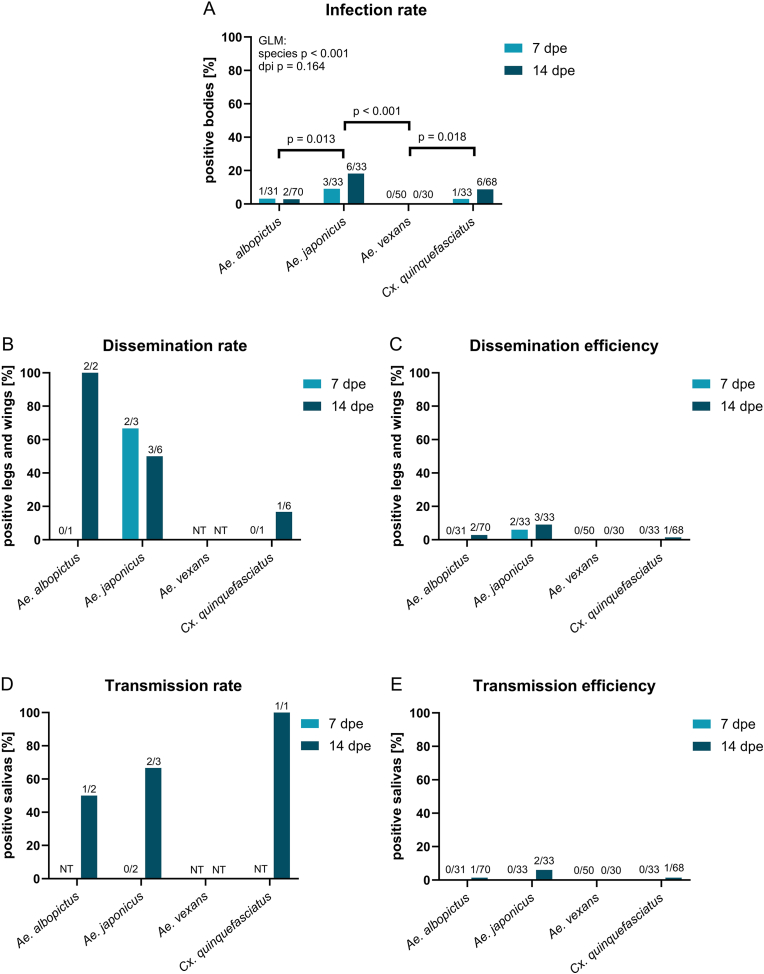
Comparison of mosquito species from Switzerland (*Aedes japonicus*, *Ae. albopictus*, *Ae. vexans*) and tropical *Culex quinquefasciatus* (reference), after exposure to Japanese encephalitis virus (JEV) and incubation at fluctuating temperatures (16–28 °C) for 7- and 14-days post-exposure (dpe). Bars represent the percentage of females (**A**) with JEV RNA in the body (**B**) with JEV RNA in legs and wings compared to infected (**C**) and all females, (**D**) with JEV RNA in the saliva compared to females with disseminated infection (**E**) and all females. Statistical analysis (GLM with logit link, Fisher’s exact for pairwise comparisons) was performed only for **A**; non-significant *P*-values not shown. No statistical tests were performed for **B**-**E** due to small sample sizes. Numbers above bars indicate positive/tested samples. Positive = Ct ≤ 35 (RT-qPCR); NT = not tested.

*Aedes albopictus*, *Ae. japonicus*, and *Cx. quinquefasciatus* were successfully infected with JEV after 7 and 14 days of incubation under fluctuating temperatures (16–28 °C). In contrast, *Ae. vexans* was not susceptible to JEV infection, with none of the 80 blood-fed mosquitoes testing positive in the RT-qPCR assay. The IR was species-dependent (GLM, LR *χ*^2^ = 16.715, *df* = 3, *P* < 0.001), whereas the duration of incubation (7 *vs* 14 dpe) had no significant effect on the IR (GLM, LR *χ*^2^ = 1.934, *df* = 1, *P* = 0.164) (Fig. 2A).

Among the different species, *Ae. japonicus* exhibited the highest IR, with 9 out of 66 (13.6%) mosquitoes testing positive for JEV RNA in the body. This was significantly higher than the IR of *Ae. vexans* (Fisher’s exact test, *P* < 0.001) (non-infected) and *Ae. albopictus*, with 3 out of 101 (3.0%) blood-fed females found infected (Fisher’s exact test, *P* = 0.013). Despite being a reference species, only 7 of the 101 (6.9%) *Cx. quinquefasciatus* tested positive for JEV under the chosen temperature conditions, which was not significantly different from *Ae. albopictus* and *Ae. japonicus* (Fig. 2A).

### 3.2 Dissemination rate and efficiency

Under our experimental conditions, *Ae. japonicus* showed disseminated infection as early as 7 dpe, with JEV RNA present in the legs and wings of 2 out of 3 (66.7%) infected females (Fig. 2B) and a total of 33 blood-fed females analysed (6.1%) (Fig. 2C). On the contrary, neither *Ae. albopictus* nor *Cx. quinquefasciatus* tested at 7 dpe were positive for JEV RNA in their legs and wings (Fig. 2B and C). Disseminated infection at 14 dpe was detected in all mosquito species except for *Ae. vexans*, which were not examined for dissemination due to the absence of JEV RNA in their bodies (Fig. 2B and C).

### 3.3 Transmission rate and efficiency

None of the mosquito species examined was identified as capable of transmitting JEV at 7 dpe. At 14 dpe, *Ae. japonicus* exhibited the highest TR and TE among all species tested, with 2 positive saliva samples out of 3 mosquitoes with disseminated infection (66.7%) (Fig. 2D) and out of a total of 33 blood-fed females analysed (6.1%) (Fig. 2E).

### 3.4 Quantification

**Fig. 3:**
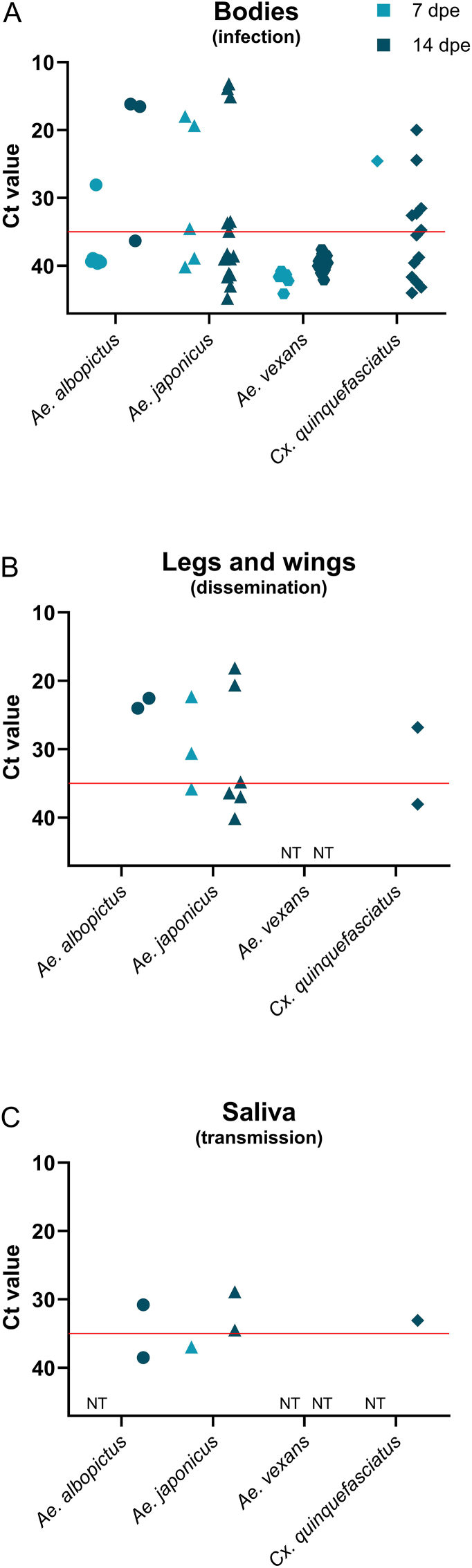
Cycle of threshold (Ct) values indicating detection of Japanese encephalitis virus (JEV) RNA by RT-qPCR in mosquito bodies (**A**), legs and wings (**B**), and saliva (**C**). Each data point represents the mean Ct-value of technical duplicates. Ct-values are presented on a reversed scale, where lower values correspond to higher viral loads. Data are grouped by mosquito species: *Aedes albopictus* (dots)*, Ae. japonicus* (triangles)*, Ae. vexans* (hexagons), and *Culex quinquefasciatus* (diamonds), as well as incubation periods of 7 and 14 days under a fluctuating temperature regime (16–28 °C) after exposure to JEV, expressed as days post-exposure (dpe). The red horizontal line marks the positive cut-off value (Ct ≤ 35). Samples without detectable JEV RNA (Ct = 0.00) are excluded from the graph. NT = not tested.

All blood-fed females collected immediately after exposure to the infectious blood meal (Day 0) tested positive for JEV RNA, with Ct-values ranging from 27.61 to 31.46 (Supplementary file 1: Table S1). Ct-values of mosquito body samples ranged widely from 13.18 to 44.77 (Fig. 3A), while Ct-values of legs and wings samples ranged from 18.12 to 40.11 (Fig. 3B). Notably, two *Ae. japonicus* body samples (Ct = 17.98 and 19.34), along with corresponding legs and wings (Ct = 22.31 and 30.57), collected at 7 dpe, showed significantly lower Ct-values compared to other mosquito species at the same time point (Fig. 3A and B) as well as to individuals of the same species tested on Day 0 (Supplementary file 1: Table S1). Furthermore, some samples of bodies, legs and wings from *Ae. albopictus*, *Ae. japonicus*, and *Cx. quinquefasciatus* collected at 14 dpe and one *Cx. quinquefasciatus* body sample (Ct = 24.54) collected at 7 dpe also showed considerably lower Ct-values in comparison to those tested at Day 0. Collectively, these findings suggest active viral replication in these species (Supplementary file 1: Tables S1 and S2).

In contrast, *Ae. vexans* body samples had Ct-values only as low as 37.57, which exceeded the positivity threshold of 35; thus, none were classified as positive (Fig. 3A). Despite this, *Ae. vexans* individuals collected on Day 0 had Ct-values between 27.61 and 29.75 (Supplementary file 1: Table S1), confirming successful ingestion of the virus and suggesting that little to no viral replication occurred in this species during the incubation period.

Saliva samples testing positive for JEV RNA had Ct-values of 30.77 (*Ae. albopictus*), 28.89 and 34.46 (*Ae. japonicus*), and 33.05 (*Cx. quinquefasciatus*). One *Ae. albopictus* saliva sample (Ct = 38.46, 14 dpe) and one *Ae. japonicus* sample (Ct = 36.94, 7 dpe) exceeded the positivity threshold and were therefore classified as negative (Fig. 3C).

## 4 Discussion

Our study demonstrates that *Ae. albopictus* and *Ae. japonicus* collected in Switzerland are competent vectors for JEV GI-b. After 14 days of incubation under a fluctuating temperature regime characteristic for Central European summers, the virus successfully infected, disseminated, and reached the saliva of these mosquito species. However, the observed transmission efficiencies were low, at 1.4% and 6.1% respectively, and were comparable to that observed in the tropical, reference species *Culex quinquefasciatus* (1.5%) in our experimental settings. In contrast, field-caught *Ae. vexans* was refractory to JEV infection. These findings suggest a potential role of *Aedes* species in JEV ecology in temperate regions alongside primary *Culex* vectors.

Clear differences in vector competence were observed among species. Field-caught *Ae. japonicus*, an invasive mosquito widely established in Central Europe, exhibited the highest IR and was the only species to develop a disseminated infection as early as 7 dpe, preceding *Cx. quinquefasciatus*. However, no saliva samples tested positive at this early stage. Similarly, Takashima and Rosen (1989) confirmed *Ae. japonicus* infection with JEV at 7 dpe, with slower viral growth at 20 °C compared to 28 °C; however, they did not test for transmission at this time point. In contrast, Faizah et al. (2020) isolated JEV GI from the saliva of *Ae. japonicus* already after 7 days of incubation at a constant 27 °C. Further investigation is needed to confirm the transmission capacity of *Ae. japonicus* during the early stage of JEV infection. Despite its demonstrated vector competence in laboratory settings, the role of *Ae. japonicus* in field transmission remains unconfirmed. This mosquito is known for its opportunistic feeding behaviour on a wide range of hosts, including pigs, humans, and birds (Schönenberger et al., 2016; Cebrián-Camisón et al., 2020). Therefore, the absence of JEV field isolates may be attributed to limited surveillance efforts targeting this species, as it is not considered a primary vector, or its low abundance in areas near JEV-amplifying hosts, such as wetlands, rice paddies, or pig farms in endemic regions, where *Culex* species are typically dominant (Seo et al., 2013; Su et al., 2014; Kim et al., 2015; He et al., 2021).

The absence of JEV infection in *Ae. vexans*, a native European floodplain mosquito, contrasts with sporadic reports of virus isolation from this species in tropical regions (Su et al., 2014). Yet, in temperate South Korea, where *Ae. vexans* is a dominant mosquito species, it consistently tested negative for JEV, even when the virus was detected in other mosquito species collected from the same sites, including animal farms, wetlands, and urban areas with reported human cases (Seo et al., 2013; Kim et al., 2015). Laboratory studies on *Ae. vexans* infections with JEV are limited, existing reports show varying results and utilise different JEV genotypes from the one used in our study (Hodes, 1946; Reeves and Hammon, 1946). These inconsistencies may be attributed to various factors, such as the climate zone, variations in virus strain (Schuh et al., 2013), mosquito microbiome or population genetics (Beerntsen et al., 2000; Zhang et al., 2024).

Our study demonstrated that *Ae. albopictus* from southern Switzerland is a competent JEV vector, with disseminated infection and detectable virus in the saliva at 14 dpe, though the transmission efficiency was limited to 1.5%. Previous studies under constant temperature regimes have also confirmed *Ae. albopictus* as vector, for several JEV genotypes. Those studies often reported higher IR, DR and TR compared to our results under a fluctuating temperature regime (Nicholson et al., 2014; de Wispelaere et al., 2017; Liu et al., 2023; Faizah et al., 2025). Viral dissemination in *Ae. albopictus* has been observed at a constant 20 °C as early as 7 dpe for JEV GIII (Liu et al., 2023), with virus RNA detected in the saliva at 30 °C (Liu et al., 2023) and 28 °C for JEV GIV (Faizah et al., 2025). In contrast, we detected no dissemination or transmission at 7 dpe. Notably, in the study of Hernández-Triana et al. (2022), *Ae. albopictus* from Italy showed no vector competence for JEV GIII even after 14 days at 25 °C. However, the mosquito generation used in that study is unknown. While sporadic field detections suggest a possible role of *Ae. albopictus* in JEV ecology (Su et al., 2014), its impact remains uncertain. This mosquito species feeds on both mammals and birds, showing a preference for humans. Its feeding behaviour, coupled with high adaptability to urban environments, has established it as an important vector for several zoonotic arboviruses (Cebrián-Camisón et al., 2020; Fikrig et al., 2023). Their involvement in the JEV transmission cycle will depend on the presence of infected reservoir hosts and local mosquito population dynamics.

JEV had often been isolated from *Culex quinquefasciatus* mosquitoes in endemic regions (Nitatpattana et al., 2005; Lindahl et al., 2013; Su et al., 2014) and was therefore included in this study as a reference species. Our results demonstrate that it can potentially transmit JEV under a fluctuating temperature regime representative of temperate climates. However, its IR and TE remained low and were comparable to those observed in secondary *Aedes* vectors. This may be attributed to exposure to fluctuating temperatures as low as 16 °C or the extended laboratory maintenance of the *Cx. quinquefasciatus* colony, which may have altered vector competence (Beerntsen et al., 2000; Bolling et al., 2015; Baltar et al., 2023). These findings are consistent with Liu et al. (2023), who used colony-derived *Cx. quinquefasciatus* maintained in the laboratory for over 40 generations and reported low dissemination and no transmission at 7 and 14 dpe with JEV genotype III at constant 20 °C. In their study IR, DR and TR increased with temperature, with transmission observed as early as 7 dpe at 30 °C. In contrast, field-collected *Cx. quinquefasciatus* from Australia infected with JEV genotype II showed no transmission at 28 °C after more than 14 dpe, whereas colony-derived mosquitoes under the same conditions did transmit the virus (Van Den Hurk et al., 2003b). However, colony-derived *Cx. quinquefasciatus* from the USA and New Zealand, infected with JEV genotype III and incubated at a constant 24 °C, showed no viral dissemination and transmission (Kramer et al., 2011). These observations highlight the influence of geographical origin, temperature, and colony history on vector competence. Further studies are needed to understand the low IR and TE observed in our reference species under fluctuating temperatures, as well as the comparatively higher IR and earlier dissemination observed in *Ae. japonicus*.

JEV GI-b, used in our study, is currently the most abundant genotype, and phylogenetic evidence links its distribution with temperate regions, supporting the hypothesis that climate plays a key role in JEV transmission dynamics (Schuh et al., 2013, 2014). Arbovirus transmission depends on the virus overcoming physiological barriers within the mosquito, including the midgut and salivary gland barriers, to reach the saliva (Van den Eynde et al., 2022; Carpenter and Clem, 2023). Temperature plays a key role in this process, affecting viral replication and the extrinsic incubation period (EIP), which determines the time between ingestion and the presence of the virus in the saliva. For instance, Winokur et al. (2020) demonstrated that *Ae. aegypti* infected with the Zika virus had a significantly shorter EIP at 30 °C (5.1 days) compared to 21 °C (24.2 days). Moreover, Folly et al. (2021) showed that at 20 °C, JEV infection in *Cx. pipiens* was restricted to the midgut, whereas at 25 °C, the virus had disseminated throughout the mosquito body. On the other hand, low temperatures may compromise mosquito antiviral immune responses, such as RNA interference (Campbell et al., 2008; Adelman et al., 2013; Ferreira et al., 2020). This mechanism could explain why Chapman et al. (2020) observed reduced IR for JEV GII in *Culiseta annulata* at higher temperatures, and Hug et al. (2024) found that *Ae. aegypti* infected with the Sindbis virus exhibited a preference for warmer temperatures during the period associated with virus dissemination.

The impact of temperature fluctuations also remains poorly understood. Van den Eynde et al. (2023) reported reduced DR of JEV GIII in *Cx. pipiens* exposed to fluctuating temperatures compared to a constant 25 °C. Similarly, Lambrechts et al. (2011) found that *Ae. aegypti* exposed to the dengue virus were less susceptible to infection under large temperature amplitudes. However, Carrington et al. (2013) observed that large temperature fluctuations around low mean temperatures enhanced viral dissemination compared to constant low temperatures, suggesting that, under certain conditions, temperature fluctuations may promote rather than inhibit vector competence. In our study, with temperatures fluctuating between 16 and 28 °C, the EIP was extended compared to studies conducted under constant temperatures with the same mosquito species (Faizah et al., 2020, 2025; Liu et al., 2023). While infection rates remained relatively low, the ability of *Ae. albopictus* and *Ae. japonicus* to support viral dissemination and transmission under these conditions suggests that temperature fluctuations did not prevent the virus from overcoming key physiological barriers in the mosquito.

A limitation of this study is the sole use of RT-qPCR for virus detection in saliva, as this method detects viral RNA both associated and not associated with infectious virus particles. While the presence of viral RNA in saliva indicates that the virus replicated in the mosquito and crossed both the midgut and salivary gland barriers, suggesting potential for transmission, confirmation of infectious virus presence in the saliva requires cell-based assays. Previous studies that employed both RT-qPCR and cell-based methods have demonstrated that positive RT-qPCR results generally correlate with the presence of infectious virus particles, as verified by cell culture assays (Hameed et al., 2019; Faizah et al., 2020; Folly et al., 2021).

## 5 Conclusions

Our findings provide new insights into the potential for JEV establishment in temperate regions, demonstrating that *Ae. albopictus* and *Ae. japonicus* from Switzerland are competent vectors under ecologically relevant temperature regimes. While traditionally considered secondary vectors, their ability to sustain infection, support viral dissemination, and transmit JEV suggests that they could contribute to virus circulation if JEV were introduced into Europe. The early dissemination observed in *Ae. japonicus* and its widespread presence in Central Europe highlight the need for further investigation into its potential role in JEV ecology. Monitoring the distribution and feeding behaviour of these mosquitoes, particularly in proximity to JEV reservoir hosts such as pigs and water birds, will be essential for evaluating the risk of transmission. Additionally, our findings reinforce the importance of considering fluctuating temperatures when evaluating the vector competence of mosquitoes from temperate regions. Public health authorities should integrate these factors into JEV emergence risk assessments, recognising the expanding presence of invasive mosquito species and their possible role in virus transmission.

## CRediT authorship contribution statement

**Anna M. Ciećkiewicz:** Investigation, Data curation, Formal analysis, Visualization, Writing – original draft. **Julia Ettlin:** Funding acquisition, Investigation, Methodology, Writing – review & editing. **Eva Veronesi:** Conceptualization, Funding acquisition, Methodology, Supervision, Writing – review & editing. **Andrea Marti:** Resources, Methodology, Writing – review & editing. **Obdulio Garcia-Nicolas:** Resources, Methodology. **Jeannine Hauri:** Investigation, Methodology. **Artur Summerfield:** Conceptualization, Funding acquisition, Resources, Methodology, Supervision, Writing – review & editing. **Alexander Mathis:** Conceptualization, Funding acquisition, Methodology, Supervision, Writing – review & editing. **Niels O. Verhulst:** Methodology, Supervision, Writing – review & editing.

## Ethical approval

Not applicable.

## Statement on the use of AI-assisted technologies

While preparing this article, the authors used OpenAI’s ChatGPT to correct grammatical errors and improve readability. After using this tool, the authors reviewed and edited the content as needed. The authors take full responsibility for the content of the published article.

## Funding

This project was financed by the 10.13039/100000001Swiss National Science Foundation (grant 192498) and the UZH Candoc Grant 2022. We highly acknowledge the Swiss Federal Food Safety and Veterinary Office as the sponsor of the Swiss National Centre for Vector Entomology. *Culex quinquefasciatus* mosquitoes were provided *via* the “10.13039/100018695Research Infrastructures for the control of vector-borne disease (Infravec2)”, which has received funding from the European Union’s Horizon 2020 Research and Innovation Programme under grant agreement No. 731060.

## Declaration of competing interests

The authors declare that they have no known competing financial interests or personal relationships that could have appeared to influence the work reported in this paper.

## Data Availability

Data supporting the conclusions of this article are included within the article and its supplementary files.
