# Supplementary material for: Vector competence of European Aedes mosquito species for Japanese encephalitis virus under fluctuating temperature conditions
Source: Curr Res Parasitol Vector Borne Dis. 2025 Jul 31;8:100302. doi: 10.1016/j.crpvbd.2025.100302 (PMC12344973; doi:10.1016/j.crpvbd.2025.100302)
Supplement: Multimedia component 1 [file mmc1.pdf]

Vector competence of European Aedes mosquito species for Japanese encephalitis virus under fluctuating temperature conditions

Anna M. Ciećkiewicz, Julia Ettlin, Eva Veronesi, Andrea Marti, Obdulio Garcia-Nicolas, Jeannine Hauri, Artur Summerfield, Alexander Mathis, Niels O. Verhulst

Supplementary file 1

Supplementary Table S1. Cycle of threshold (Ct) values indicating detection of Japanese encephalitis virus (JEV) RNA by RT-qPCR in blood-fed mosquitoes collected on the day of exposure to the infectious blood meal (Day 0). The Ct values are the mean of technical duplicates, with lower values corresponding to higher viral loads. Data are grouped by mosquito species (*Ae. japonicus*, *Ae. albopictus*, *Ae. vexans*, and *Cx. quinquefasciatus*).

Mosquito species	Ct Mean
Ae. albopictus	28.31
Ae. albopictus	28.36
Ae. albopictus	28.66
Ae. albopictus	29.74
Ae. albopictus	30.65
Ae. albopictus	30.80
Ae. japonicus	28.13
Ae. japonicus	29.67
Ae. japonicus	30.25
Ae. japonicus	30.48
Ae. japonicus	31.11
Ae. vexans	27.61
Ae. vexans	28.94
Ae. vexans	29.75
Cx. quinquefasciatus	28.88
Cx. quinquefasciatus	29.25
Cx. quinquefasciatus	29.92
Cx. quinquefasciatus	30.33
Cx. quinquefasciatus	30.79
Cx. quinquefasciatus	30.99
Cx. quinquefasciatus	31.11
Cx. quinquefasciatus	31.29
Cx. quinquefasciatus	31.44
Cx. quinquefasciatus	31.46

Supplementary Table S2. Cycle of threshold (Ct) values indicating detection of Japanese encephalitis virus (JEV) RNA by RT-qPCR in mosquito body parts and saliva at 7 and 14 days post-exposure (dpe) to the infectious blood meal. The Ct values are the mean of technical duplicates, with lower values corresponding to higher viral loads. Data are grouped by mosquito species (*Ae. japonicus*, *Ae. albopictus*, *Ae. vexans*, and *Cx. quinquefasciatus*).

Ae. albopictus		*Ae. japonicus*		*Ae. vexans*		*Cx. quinquefasciatus*	
7 dpe	14 dpe	7 dpe	14 dpe	7 dpe	14 dpe	7 dpe	14 dpe
Ct of bodies							
28.04	16.14	17.98	13.18	40.76	37.57	24.54	19.96
38.89	16.52	19.34	13.87	41.25	38.00		24.41
39.08	36.30	34.50	15.10	41.26	38.08		31.52
39.22		38.86	33.44	41.58	38.21		32.27
39.28		40.13	33.66	42.17	38.46		32.56
39.33			34.91	44.09	38.66		34.72
39.42			38.23		38.80		35.46
39.61			38.53		38.81		38.72
			38.96		38.88		39.54
			38.99		39.12		41.62
			41.05		39.23		42.35
			41.30		39.29		43.13
			41.63		39.51		43.97
			42.98		39.83		
			44.77		39.84		
					40.00		
					40.05		
					40.16		
					40.23		
					40.30		
					40.58		
					40.91		
					40.99		
					42.05		
Ct of legs and wings							
	22.52	22.31	18.12				26.78
	23.98	30.57	20.61				38.01
		35.79	34.77				
			36.37				
			36.92				
			40.11				
Ct of saliva							
	30.77	36.94	28.89				33.05
	38.46		34.46				

Time	Temperature [°C]
00:00	19
03:00	17
05:00	16
06:00	18
07:00	20
08:00	23
09:00	24
11:00	27
13:00	28
16:00	26
19:00	24
21:00	22

Supplementary Table S3. The fluctuating temperature regime used during mosquito incubation post exposure to JEV.
